# A Comprehensive Insight into Binding of Hippuric Acid to Human Serum Albumin: A Study to Uncover Its Impaired Elimination through Hemodialysis

**DOI:** 10.1371/journal.pone.0071422

**Published:** 2013-08-09

**Authors:** Nida Zaidi, Mohammad Rehan Ajmal, Gulam Rabbani, Ejaz Ahmad, Rizwan Hasan Khan

**Affiliations:** Interdisciplinary Biotechnology Unit, Aligarh Muslim University, Aligarh, Uttar Pradesh, India; University of Hyderabad, India

## Abstract

Binding of hippuric acid (HA), a uremic toxin, with human serum albumin (HSA) has been examined by isothermal titration calorimetry (ITC), differential scanning calorimetry (DSC), molecular docking, circular dichroism (CD) and fluorescence spectroscopy to understand the reason that govern its impaired elimination through hemodialysis. ITC results shows that the HA binds with HSA at high (*K*_b_ ∼10^4^) and low affinity (*K*_b_ ∼10^3^) sites whereas spectroscopic results predict binding at a single site (*K*_b_∼10^3^). The HA form complex with HSA that involves electrostatic, hydrogen and hydrophobic binding forces as illustrated by calculated thermodynamic parameters. Molecular docking and displacement studies collectively revealed that HA bound to both site I and site II; however, relatively strongly to the later. Esterase-like activity of HSA confirms the involvement of Arg410 and Tyr411 of Sudlow site II in binding of HA. CD results show slight conformational changes occurs in the protein upon ligation that may be responsible for the discrepancy in van’t Hoff and calorimetric enthalpy change. Furthermore, an increase in 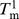
and 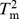
is observed from DSC results that indicate increase in stability of HSA upon binding to HA. The combined results provide that HA binds to HSA and thus its elimination is hindered.

## Introduction

Uremic toxins are the compounds which retained in the blood during kidney failure and interact negatively with the normal biological functions of the body [1]. Hippuric acid (HA) is one of these compounds that accumulates in the blood, and cause stimulation of ammoniagenesis. It is involved in development of muscular weakness in uremia as it also inhibits glucose utilization in muscles [2]–[4]. It has also been related to inhibition of organic anion secretion by the kidney [5] and transport at the blood-brain barrier [6]. Consequently, HA is a compound of pharmacological interest. It is a glycine conjugate of benzoate, which is formed primarily from aromatic amino acids by gastrointestinal flora or may be directly taken as preservatives from food and beverages [7]. In a healthy individual, concentration of HA is less than 5 mg/L but increases to values higher than 247±112 mg/L in patients with end-stage renal disease [8].

Human serum albumin (HSA) is the most abundant plasma protein, single chain, nonglycosylated polypeptide of 66.5 kDa. It is composed of three homologous, predominantly helical domains I–III, each of which contains two subdomains A and B [9]. HSA has one tryptophan residue, Trp214, located in subdomain IIA [10], [11]. The principal regions of ligand binding to HSA are located in hydrophobic cavities in subdomains IIA and IIIA, which are consistent with Sudlow sites I and II, respectively [12]. These binding sites underline the exceptional ability of HSA to interact with many organic and inorganic molecules, thereby making this protein an important regulator of the pharmacokinetic behavior of many drugs as well as intercellular fluxes [13]. In body, it also binds to HA [2] and thus elimination of HA through hemodialysis is only 64% [14], [15]. However, there is paucity of information on its binding mechanism to HSA. Consequently, it is necessary to investigate the binding energetic, amino acid involved in binding of HA to HSA to explore its binding mechanism in the body. So, the scope of this work is to evaluate these in details by studying the binding energetic using steady state fluorescence spectroscopy and isothermal titration calorimetry. Binding sites is determined by displacement studies whereas estimation of amino acid involved in binding, by molecular docking and esterase-like activity of HSA toward *p*-NPA. Thermal stability in presence of HA is determined using differential scanning calorimetry.

## Materials and Methods

### Materials and Sample Preparation

Human serum albumin (A1887; >96%), warfarin (A2250; >98%), phenylbutazone (P8386; >99%), and *p*-nitrophenyl acetate (N8137; >99%) were procured from Sigma Aldrich. Hippuric acid (free acid, crystalline; >99%) was from Himedia. The number in the parenthesis corresponds to the purity of the compounds. All other reagents were of analytical grade. HSA and drug solutions were prepared in 20 mM sodium phosphate buffer (pH 7.4). HSA was passed through Sephacryl-S200 gel filtration column, dialyzed, and its concentration was estimated spectrophotometrically using 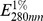 = 5.3. All drug solutions were prepared by weight/volume (w/v).

### Steady State Fluorescence Quenching Measurements

Fluorescence emission spectra were recorded in range of 300–400 nm on a Shimadzu 5301PC fluorescence spectrophotometer equipped with water circulator (Julabo Eyela) at excitation wavelength of 295 nm. Both the excitation and emission slits were set at 3 nm. The titration of the HA (0–10 µM) to HSA (5 µM) solution was carried out at 25, 30, and 37°C. Respective blanks were subtracted. The fluorescence data were analyzed according to the Stern–Volmer equation to obtain Stern–Volmer quenching (*K*_sv_) and bimolecular rate constant (*k*_q_) [16]:
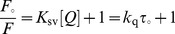
(1)where, *F*_o_ and *F* are the fluorescence intensities in the absence and presence of quencher (HA), and *τ*_o,_ is the average integral fluorescence life time of tryptophan (∼ 5.71×10^−9 ^s) [17]. Furthermore if a quencher, *Q,* form complex, *Q*_n_ *B,* with protein *B* and have ‘*n*’ multiple (equivalent and independent) binding sites then quenching reaction is often represented as:
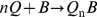
(2)and its binding constant (Kb ) is given by equation 3:[18]



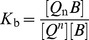
(3)If the total amount of protein (with and without bound Q) is [*B*_o_], then
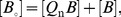
(4)

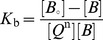
(5)where [*B*] is the concentration of the unbound protein. The fluorescence intensity is proportional to the protein concentration as described below:
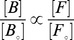
(6)then, relationship between the fluorescence intensity and unbound protein is expressed as:



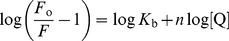
(7)So, if the formation of a nonfluorescent fluorophore–quencher complex occurs, then, the value of *K*_b_ can be obtained by using equation 7.

Furthermore, change in standard Gibbs free energy (Δ*G°*) was obtained using Gibbs-Helmholtz equation:
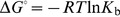
(8)whereas, change in standard enthalpy (Δ*H* ^o^) and entropy (Δ*S* ^o^) were determined from the van’t Hoff equation, if Δ*H* ^o^ do not vary significantly over the temperature range studied.
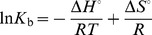
(9)where *R* (1.987 cal mol−1K−1) is gas constant and T is the absolute temperature (K)

### Binding Displacement Measurement Using Site Markers

Different site markers warfarin (WAR) for site I and diazepam (DIA) for site II [19], [20] were used for performing displacement experiments. The titration of HA were carried out to the solution having protein and site marker in the ratio of 1∶1. The fluorescence emission spectra were recorded as mentioned in fluorescence measurements and the binding constant values of drug–protein–marker were evaluated using Stern–Volmer equation.

### Isothermal Titration Calorimetric Measurements (ITC)

The VP-ITC titration microcalorimeter (MicroCal Inc., Northampton, MA) were used to gain insight into the energetics of the binding of HA to HSA at 25, 30, and 37°C. Prior to the titration experiment, all solutions were degassed properly on a thermovac. The 1.44 mL sample and reference cell of the calorimeter were loaded with HSA and 20 mM sodium phosphate buffer (pH 7.4), respectively. The HSA (25 µM) was titrated with HA (1.928 mM) using a 288 µL injection syringe stirring at 307 rpm. Equal volumes of HA solutions (10 µL) were injected into the sample cell containing HSA over 20 s with an interval of 180 s between injections. The reference power was set at 16 µcal s^−1^. The heat associated with each injection was observed as a peak that corresponds to the power required to keep the sample and reference cells at identical temperatures and the data were plotted as integrated quantities. Control experiments were performed by titrating HA into the same buffer to obtain the heats of ligand dilution. Heats of dilution for the ligand and protein were subtracted from the integrated data before curve fitting. The data were fitted and analyzed with a sequential model of two binding sites using Origin 7.0 provided with the MicroCal instrument. Association constant (*K*_b_) and standard enthalpy change (Δ*H*°) were directly obtained after fitting while Δ*G°* was calculated from equation 8. The Δ*S* ^o^ was calculated using the equation:
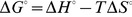
(10)and change in specific heat capacity can be calculated from the equation:



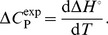
(11)Further the standard van’t Hoff enthalpy 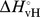 at each temperature was calculated using equation:
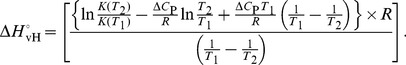
(12)here, *K*(*T_1_*) and *K*(*T_2_*) are the values of binding constant at temperatures *T*_1_ and *T*_2_ respectively.

### Circular Dichroism Spectroscopic Measurements

To monitor the secondary and tertiary structural change of protein upon interaction with HA, CD spectra of HSA were collected in far (200−250 nm) and near-UV (250–320 nm) at molar ratio of 1∶0, 1∶5, 1∶10 and 1∶15 in a JASCO-J815 spectropolarimeter equipped with a Peltier-type temperature controller at 25°C. The CD spectra were collected with 20 nm/min scan speed and a response time of 2 s. The HSA concentration and pathlength were 5 µM and 0.1 cm, respectively, for far UV CD measurement whereas 15 µM and 1 cm, respectively, for near UV CD measurement. Respective blanks were subtracted. The results were expressed as MRE (mean residue ellipticity) in deg cm^2^ dmol^−1^, which is given by:
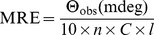
(13)where Θ_obs_ is the observed ellipticity in millidegrees, *C* is the concentration of protein in mol/l, *l* is the length of the light path in centimeters and *n* is the number of peptide bonds.

### Differential Scanning Calorimetric Measurements (DSC)

The differential scanning calorimetric measurements were carried out using VP-DSC microcalorimeter (MicroCal, Northampton, MA). The buffer and protein solutions were degassed under mild vacuum prior to the experiment. Samples were prepared in 20 mM sodium phosphate buffer, pH 7.4. The DSC measurements of HSA (18 µM) in the presence of different ratios of HA viz. 1∶0, 1∶5, and 1∶10 were performed from 25 to 90°C at a scan rate of 0.5°C/min. Data were analyzed using Origin software provided with the instrument to obtain the temperature at the midpoint of the unfolding transition (*T*_m_) and calorimetric enthalpy (Δ*H*°).

### Effect of HA Binding on Esterase-Like Activity of HSA

Drug site II (Subdomain III A) of HSA possessed esterase-like activity toward *p*-nitrophenyl acetate (*p*-NPA) [21]. Thus, the reaction of *p*-NPA with HSA in the absence and presence of HA (i.e. 0–45 µM) was followed on Perkin-Elmer Lambda 25 double beam UV–vis spectrophotometer attached with Peltier temperature programmer-1 (PTP–1) at 405 nm by monitoring the appearance of yellow product *p*-nitrophenol for 1 min at 25°C. The molar extinction coefficient of *p*-nitrophenol was taken as 17800 M^−1 ^cm^−1^. The reaction mixture contained 15 µM HSA whereas *p*-NPA varied from 0 to 600 µM in 20 mM sodium phosphate buffer (pH 7.4). The control (in the absence of HSA) was also taken in consideration. Kinetic constants were obtained using Graph-Pad Prism, version 5.0, software by fitting the initial rates to Michaelis–Menten equation:
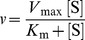
(14)where ν, *V*_max_, *K*_m_, and [S] represent the initial reaction velocity, maximum velocity, Michaelis–Menten constant, and molar concentration of substrate, respectively. Further, inhibitor constant (*K*_i_ ) was calculated from the equation:
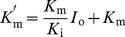
(15)where, 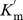, is the apparent Km in presence of competitive inhibitor concentration Io.

### Molecular Docking

The SDF format for 3D structure of HA was downloaded from PubChem database (CID 464) and crystal structure representing HSA was extracted from Protein Data Bank (PDB: 1AO6). Molecular docking simulation of HA to HSA was performed with Autodock Vina program [22]. Autodock was used to evaluate ligand binding energies over the conformational search space using Lamarckian genetic algorithm. The residues falling within 5 Å of the two different binding sites of HSA (site I & site II) were extracted and combined to define the binding site residues. Default docking parameters were used. We considered only the minimum energy conformation state of ligand bound protein complex in our study out of ten generated binding modes. The hydrogen bonding and hydrophobic interactions between ligand and protein were calculated by Accelrys DS Visualizer 2.0 [23] and figure was visualized with Chimera 1.7 [24]. By using the equation 8, the binding constants (*K*_b_) for protein-ligand interactions were calculated from the obtained free energy changes of docking.

### Statistical Analysis

All determinations were triplicates, and mean values and standard deviations were calculated, wherever applicable, using SPSS 16.0 programme for windows.

## Results and Discussion

### Steady State Fluorescence Quenching Measurements

**Figure 1:**
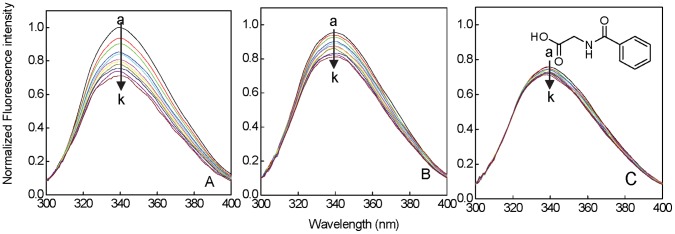
Normalized fluorescence emission spectra of HSA in the presence of different concentrations of HA at (A) 25°C, (B) 30°C, and (C) 37°C. a-k: 0—10 µM of HA at increments of 1 µM. The inset corresponds to the molecular structure of HA.

**Figure 2:**
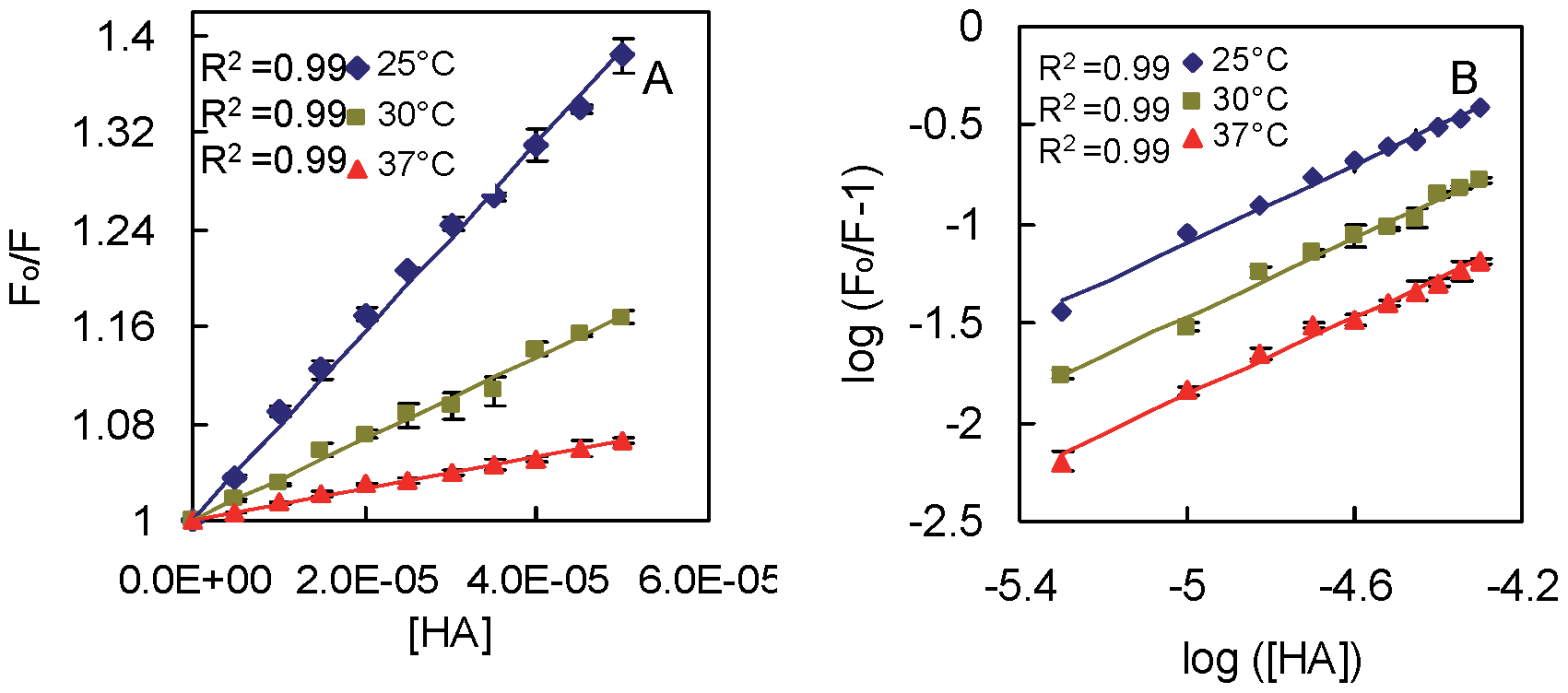
Stern—Volmer (A) and log[(F_0_-F)/F] versus log[HA] (B) plot at different temperatures. Protein (5 µM) was excited at 295 nm.

The aromatic fluorophores, tryptophan, tyrosine, and phenylalanine are very sensitive to their microenvironment and thus used for studying conformational changes associated with drug protein binding. However, tryptophan contributes maximumally to the fluorescence [25]. The fluorescence intensity of HSA decreases with gradual addition of HA at 25, 30, and 37°C as shown in Figure 1. Thus to investigate the mechanism of quenching, the fluorescence quenching data at 25, 30, and 37°C were analyzed according to equation 1. The values of *K*_sv_ and *k*_q_ obtained from Stern-Volmer plot (Figure 2A) and are listed in Table 1. It can be seen that, the values of *K*_sv_ decreases with increasing temperature and *k*_q_ was founds to be 10 times greater than the 2×10^10^ M^−1 ^s^−1^, a maximum scatter collision quenching constant of various quenchers with biopolymers. This shows that quenching was not initiated by dynamic diffusion but from the formation of a strong complex between HSA and HA [17]. As the quenching mechanism was determined to be static, so the binding constant, *K*_b_, can be calculated according to equation 7 from the y-axis intercept of plot of log [(*F*_0_ - *F*)/*F*] versus log [HA] (Figure 2B). The values of *K*_b_ obtained at different temperature are listed in Table 1. It can be seen that, the values of *K*_sv_ and *K*_b_ were almost same that further indicates static quenching mechanism [18]. Further, Δ*G* ^o^, Δ*H* ^o^ and Δ*S* ^o^ for the interaction between HSA and HA were calculated according to thermodynamic equation (equation 8) and van’t Hoff equation (equation 9), and the values thus obtained are shown in Table 1. For protein-drug interaction, the signs and magnitude of thermodynamic parameters (Δ*H* ^o^ and Δ*S* ^o^) can be used to determine the main forces that contribute in complex formation of protein-drug [26]. Thus, the negative Δ*H* ^o^ indicates the exothermic nature and dominant involvement of electrostatic interactions in the process of HSA-HA complex formation [27]. However, hydrogen bonding also play role as depicted from the negative signs of Δ*H* ^o^ and Δ*S* ^o^ according to Ross and Subramanian [26]. Furthermore, Δ*H* ^o^ contributes maximally rather than Δ*S* ^o^ to Δ*G* ^o^ that indicates the binding process is enthalpy driven and the decrease in entropy is due to the formation of hydrogen bonds between HA and HSA. In addition, negative sign of Δ*G* ^o^ indicates that the binding of HA with HSA is a spontaneous process. However, the obtained value of thermodynamic parameters may not necessarily the actual values as, such non–calorimetric approach to the thermodynamics has ruthless shortcomings where usually Δ*H*° is assumed to be temperature–independent. However, this is the only method to determine an estimate of Δ*H* ^o^ and Δ*S* ^o^ from the fluorescence quenching data at different temperature [28]. Furthermore, the binding affinity observed by fluorescence spectroscopy took in consideration the location of quencher, fluorophore and so measures local changes around the fluorophores associated with the optical transition [29]. Hence to overcome all these shortcomings, we have done ITC measurements that consider overall global changes [30].

**Table 1:** Binding and thermodynamic parameters of HSA-HA at different temperature obtained from fluorescence quenching experiments^a^.

T (°C)	n	K_SV_ (M^−1^)	K_b_ (M^−1^)	*k*_q_ (M^−1 ^s^−1^)	TΔS° b	ΔH° b	ΔG° b
25	0.98±0.02	(7.80±0.36) ×10^3^	(6.84±0.19 )×10^3^	(1.36±0.06 ) ×10^12^	−23.48±0.24		−5.21±0.02
30	0.99±0.01	(3.30±0.02) ×10^3^	(3.11±0.22) ×10^3^	(5.77±0.03 ) ×10^11^	−23.88±0.24	−25.15±0.20	−4.88±0.06
37	0.97±0.02	(1.31±0.08) ×10^3^	(1.04±0.01) ×10^3^	(2.29±0.01 ) ×10^11^	−24.43±0.25		−4.28±0.01

a R^2^ for all values ranges from 0.98 to 0.99.

b expressed in kcal mol^−1^

### Binding Displacement Measurement using Site Markers

Sudlow et al [12] proposed that the HSA has two major binding regions namely Sudlow’s site I and site II. Site I and II have affinity for WAR and DIA respectively. Thus these drugs were used as site specific markers of HSA. To trace the binding site of HA on HSA, the emitted fluorescence intensity data in the absence and presence of markers were calculated using Stern–Volmer equation. The *K*_sv_ value of HSA-HA was (7.80±0.36)×10^3 ^M^−1^ that decreases to (2.74±0.04)×10^3 ^M^−1^ and (2.03±0.03)× 10^3 ^M^−1^ in presence of WAR and DIA, respectively. These differences in *K*_sv_ values in absence and presence of site markers are significant enough to deduce the binding sites location as reported in literature [31], [32]. As evident from above values, the *K*_sv_ of HSA-HA decreased markedly in presence of WAR and DIA both, however, relatively more in later. It indicates competition between markers and HA for both site I and site II, however, relatively more for later. Thus, HA binds relatively more to site II as compared to site I.

### Isothermal Titration Calorimetric Measurements

**Figure 3:**
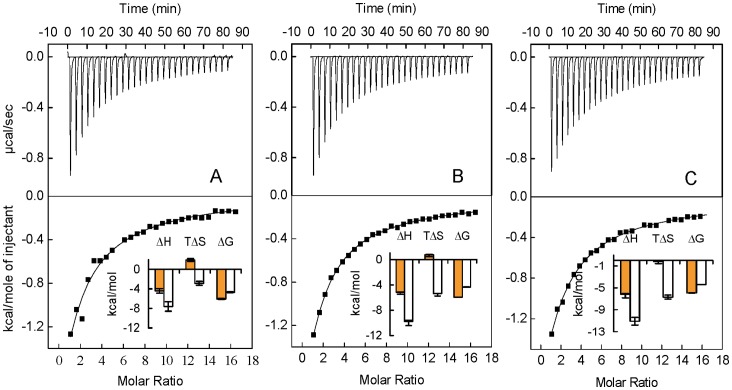
Isothermal titration calorimetry of HSA and HA interaction at different temperatures. A—C represent ITC profiles of HSA-HA system at 25, 30, and 37°C, respectively. Titration of HA with (25 µM) HSA at pH 7.4 shows calorimetric response as successive injections of ligand is added to the sample cell. The solid line represent the best nonlinear least-squares fit of sequential model of two binding site. The inset of A-C represent comparative bar distribution of Δ*H*, Δ*S* and Δ*G* obtained from ITC. Each thermodynamic parameter represented by two bars, open (low affinity site) and filled (high affinity site).

ITC was used to measure binding affinity and energetics of HA to HSA. Figure 3 shows the ITC binding isotherm of HA to HSA at 25, 30, and 37°C in which each peak in top panel represents a single injection of the drug into protein solution. Bottom panel of this figure shows an integrated plot of the amount of heat liberated per injection as a function of the molar ratio of the drug to protein. The best fits for the integrated heats was obtained using a two sites sequential binding model with the lowest *χ* ^2^. The temperature dependency of the thermodynamic binding parameters of HA to HSA obtained after fitting is summarized in Table 2. These results showed that the binding affinity is in the order of 10^4^ and 10^3^ for high and low affinity binding site respectively which decrease with increase in temperature indicating the formation of complex. The enthalpic and entropic contributions to the Gibbs free energy of binding were used to infer information regarding the mechanism of binding. It can be seen from Figure 3 (insets), that all studied temperature, the enthalpic changes for the binding of HA to both classes of binding site of HSA are all negative, which indicate that the binding process are all exothermic and involves electrostatic interactions [27]. On contrary, the entropic contributions were favourable for higher affinity binding site while unfavourable for low affinity binding site. It suggests the involvement of hydrogen bonding in binding of HA to low affinity site on HSA [26]. Whereas, negative value of Δ*G* ^o^ suggest that the formation of complex was spontaneous in nature for both set of binding sites at all three temperatures. Further, the slope of plot of Δ*H* ^o^ against *T*Δ*S* ^o^ with was found to be approximately equal to unity which indicates the enthalpy-entropy compensation effect, a common phenomenon in protein ligand interaction [33]. Besides, it was also observed that Δ*H* ^o^ varies almost linearly with examined range of temperature and so the change in heat capacity (Δ*C_P_*) was determined according to equation 11. The values of Δ*C_P_* obtained were −0.14±0.11 and −0.28±0.24 kcal mol^−1^°C^−1^ for high and low affinity site respectively. As can be seen from Table 1 and 2, value of binding affinity obtained by ITC differ from fluorescence spectroscopy that may be due to the consideration of the location of quencher and fluorophore in the later [29], [30], [34]. Not only binding affinity, but the values of Δ*H* ^o^ and *T*Δ*S* ^o^ also differ whereas the values of Δ*G* ^o^ obtained from both methods are comparable. It is due to the already discussed shortcoming of non–calorimetric approach to the thermodynamics that in this approach usually Δ*H*° is assumed to be temperature–independent as can be seen in literature [27], [31], [32], [35]. Moreover, it cannot be neglected that the evaluation of the thermodynamic parameters obtained from the spectroscopic measurements also based upon the temperature dependence of the binding affinity that may be influence by the location of quencher from the Trp214 as discussed [30]. This leads to miscalculation of temperature–dependent ΔH° and ΔS°. However, in literature non–calorimetric determination of thermodynamic parameters from fluorescence quenching data at different temperature has been exploited to get an estimate [25], [31], [32], [35]. Besides, temperature dependency of binding affinity obtained from ITC, is used to calculate the van’t Hoff enthalpy values, which do not agree well with the calorimetric enthalpies at all studied temperatures. For example, the value of van’t Hoff enthalpy obtained at 25°C obtained by using equation 12 was −11.98 and −24.69 kcal mol^−1^ at high and low affinity site respectively which differ from calorimetric enthalpies. Such difference is also reported in literature [34] and may indicate that the conformational changes are associated with the binding process which may be induced either by ligand binding or by an increase in temperature. Hence to have better understanding of conformational changes on ligand binding, we have done circular dichroism measurements.

**Table 2:** Thermodynamic parameters and association constant of HSA-HA obtained by isothermal titration calorimetry.

	T (°C)	K_b_ (M^−1^)	ΔH° a	TΔS° a	ΔG° a
High affinity site	25	(2.75±0.51)×10^4^	−4.41±0.46	1.90±0.30	−6.05±0.10
	30	(1.95±0.05)×10^4^	−5.18±0.07	0.76±0.06	−5.94±0.01
	37	(1.54±0.06)×10^4^	−6.14±0.15	−0.20±0.01	−5.93±0.02
Low affinity site	25	(2.80±0.49)×10^3^	−7.58±0.96	−2.88±0.40	−4.69±0.10
	30	(1.38±0.03)×10^3^	−9.74±0.34	−5.39±0.35	−4.35±0.02
	37	(1.29±0.07)×10^3^	−11.11±0.69	−6.69±0.36	−4.41±0.03

a expressed in kcal mol^−1.^

### Circular Dichroism Measurements

**Figure 4:**
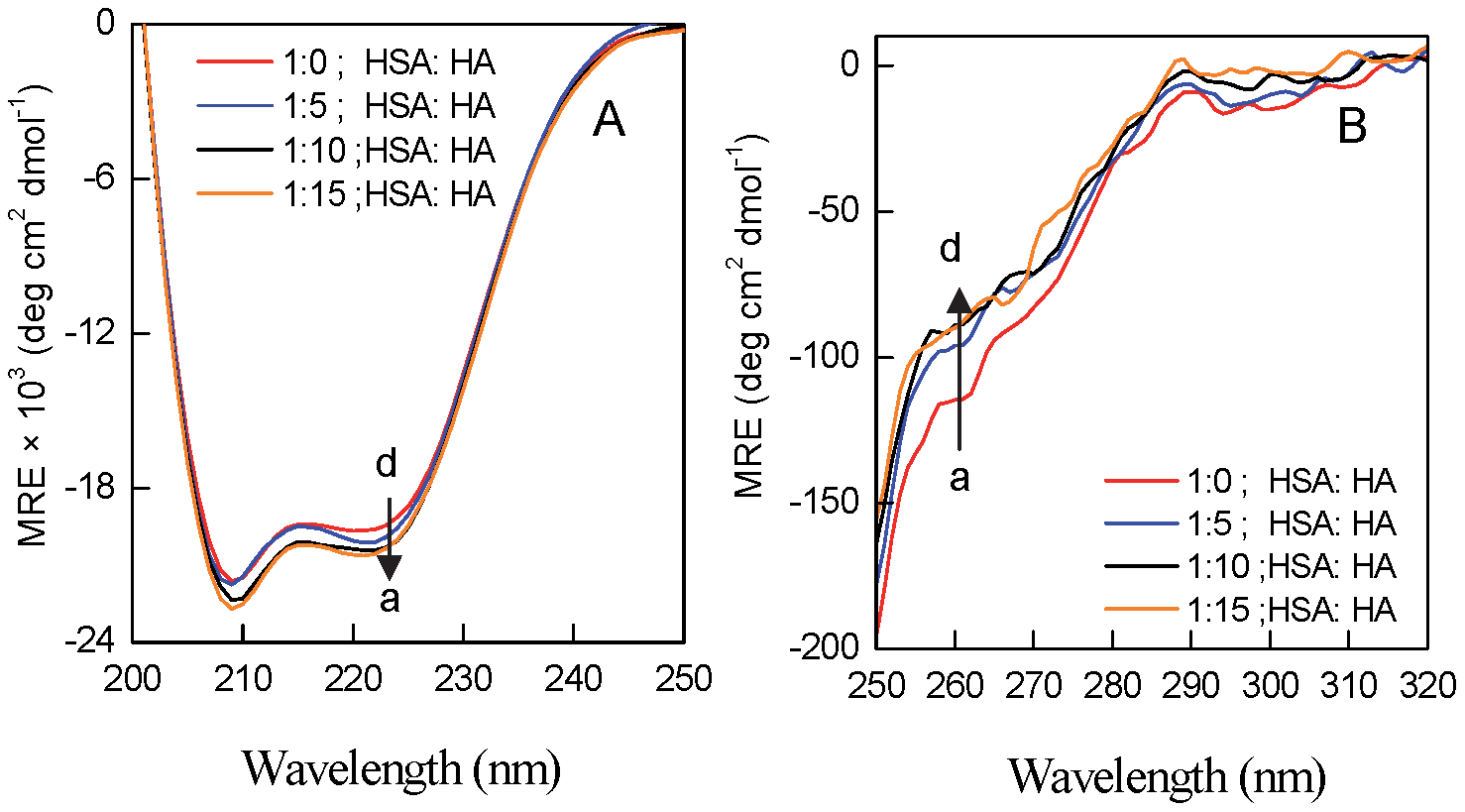
Far (A) and near (B) UV CD spectra of HSA in the presence of varying concentration of HA. a-d represent spectra of HSA: HA at molar ratio of 1∶0, 1∶5, 1∶10 and 1∶15 respectively in both (A) and (B).

The changes in secondary and tertiary structure of the HSA in presence of HA were studied in far-UV CD and near UV CD region at different molar ratio of protein to HA. Figure 4 A & B shows the far-UV CD and near UV CD spectrum of HSA in presence of [HSA]/[HA] ratio of 1∶0, 1∶5, 1∶10 and 1∶15. In the presence of HA, slight increase occur in the secondary structure of HSA as evident from the increase in two minima at 208 and 222 nm that are characterstic of α-helix [17]. However, the shape of peaks and the position of peak maximum remained almost unchanged in the presence of HA, indicating that HSA has predominantly α-helix in nature even after binding to the HA. Further as shown in Figure 4 B, near-UV CD spectra for the HSA showed two minima at 262 and 268 nm and shoulders at 279 and 290 nm, characteristics of disulphide and aromatic chromophores, which is in accordance with literature [36]. But, change in HSA spectra in presence of HA was observed that indicates the alteration of tertiary structure at different molar ratio of protein to HA. This confirms that conformational changes occur in the protein upon ligation and thus difference is observed in the values of van’t Hoff and calorimetric enthalpies obtained from ITC.

### Thermostability Measurement by Differential Scanning Calorimetry

**Figure 5:**
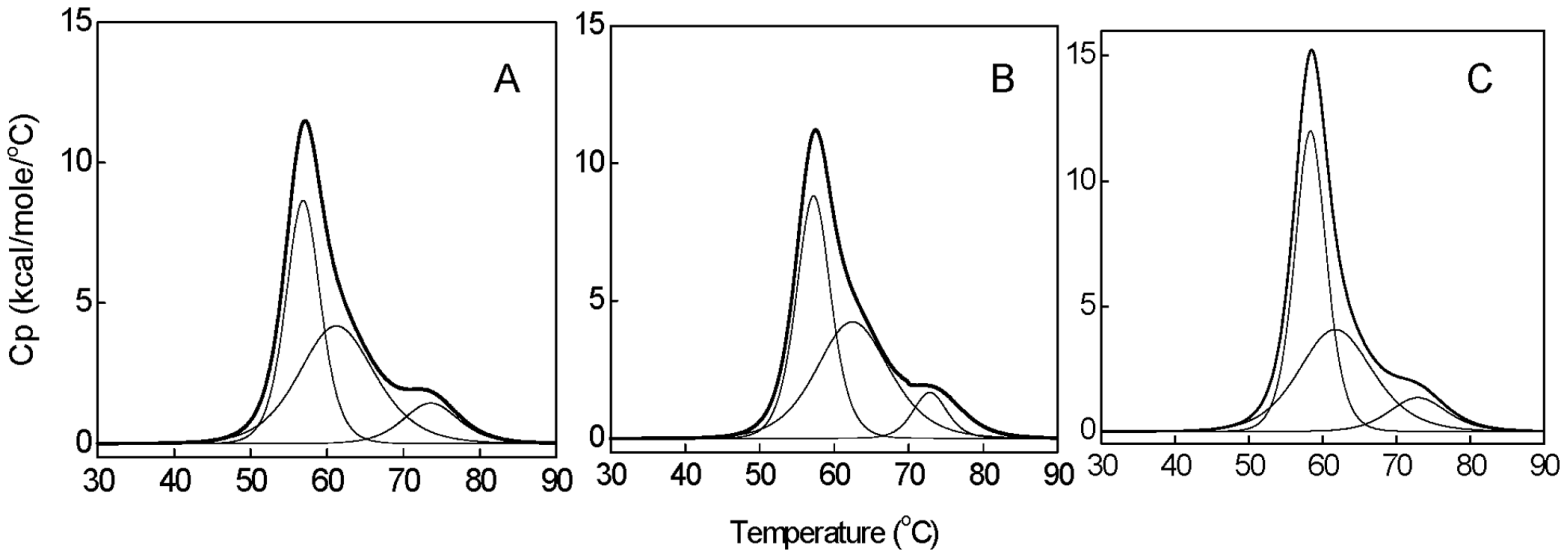
Excess heat capacity curves obtained by differential scanning calorimetry for HSA:HA in the molar ratio of (A) 1∶0, (B) 1∶5, and (C) 1∶10.

Figure 5 A–C shows the typical excess heat capacity curves for HSA:HA in the molar ratio of 1∶0, 1∶5, and 1∶10 and thermodynamic parameters obtained accompanying thermodynamic denaturation of HSA under these conditions are reported in Table 3. It is observed that the thermal unfolding of HSA is irreversible process in absence and presence of HA by reheating the samples after cooling just after the first run. Hence to minimize the kinetic factors, slower scanning rate have been chosen. The changes in the *T*_m_ and Δ*H*° of the protein in presence of ligand are the most obvious manifestation of ligand binding effects that can be estimated by DSC [37]. Thus, to confirm binding of HA to HSA, changes in the *T*_m_ and Δ*H*° have been monitored by DSC. The denaturation of HSA yielded more than one endothermic peak that reflects the domain denaturation mechanism [38]. Thus, it was deconvoluted with the assumption of three sub-transitions and each of which might be related to the links between the three structural domains of HSA. Further, it is also established that domain III melts prior to domain II, so 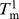
may corresponds to domain III [12], [39]. Table 3 shows 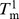, 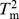 and 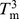
and respective Δ*H*° of native HSA that are in accordance with the literature [40]. Upon increasing molar ratio to 1∶10, the 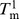 increases appreciably, 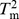 changes slightly whereas, 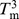 donot change at all. Besides, the increases in 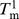 and 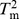 are accompanied with an increase in the value of enthalpy of unfolding, however, slightly in latter. This indicates that, binding of HA to domain III is stronger as compare to domain II whereas, negligible to domain I as higher energy is required to change from the liganded native state into the free unfolded state in case of domain III followed by domain II. Thus, HA preferential binds to the folded or native form of the HSA which causes stabilization of the folded state and hence unfolding of HSA become progressively less favorable as HA concentration increases [41], [42].

**Table 3:** Thermodynamic parameters obtained by differential scanning calorimetry.

HSA:HA	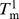 (°C)	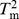 (°C)	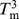 (°C)	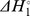 a	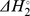 a	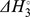 a
1∶0	56.91±0.09	61.37±0.07	73.65±0.06	52.21±0.69	55.37±0.71	13.96±0.08
1∶5	57.25±0.01	62.51±0.09	73.87±0.04	54.41±0.10	56.13±0.12	13.92±0.07
1∶10	58.36±0.08	63.83±0.02	73.96±0.03	68.47±0.80	57.37±0.60	13.90±0.09

a expressed in kcal mol^−1.^

### Measurement of Esterase-Like Activity of HSA in Presence of HA

**Figure 6:**
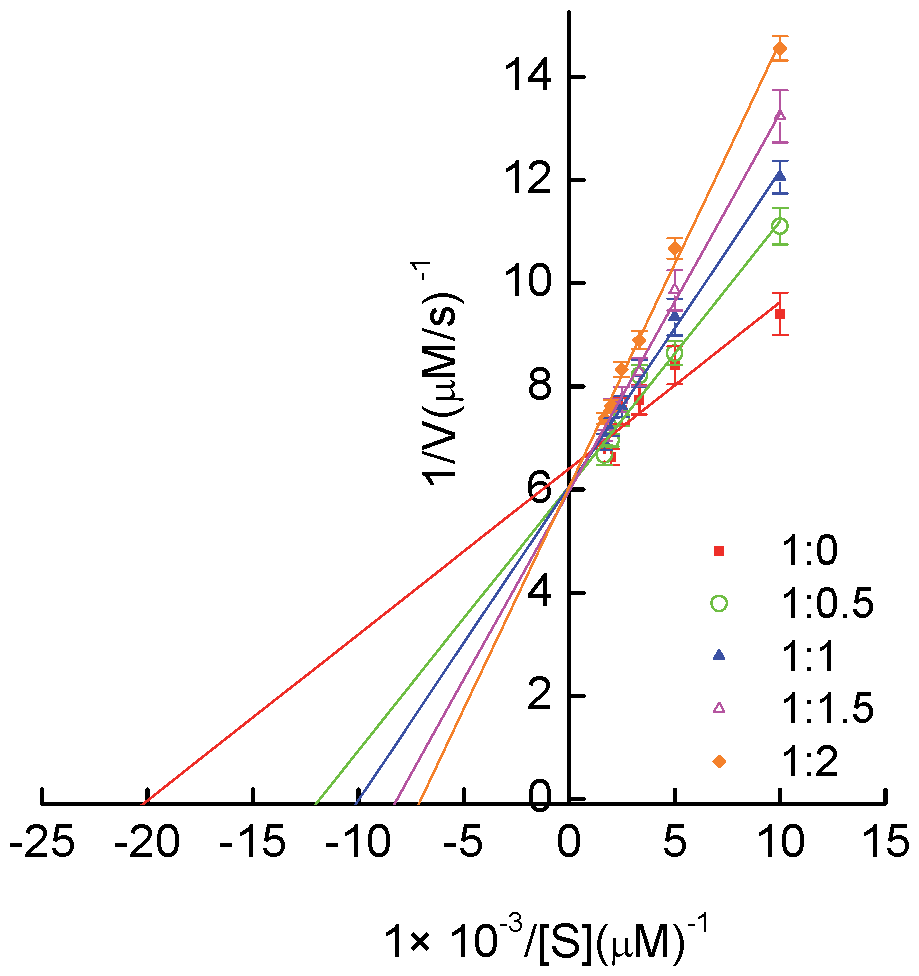
Lineweaver-Burk plots of reaction velocity versus substrate concentration for enzyme kinetics of HSA in absence and presence of HA. The molar ratio of HSA:HA examined are 1∶0, 1∶0.5, 1∶1, 1∶1:5, and 1∶2.

The Arg410 and Tyr411, crucial amino acid residue present in the centre of drug binding site II of HSA are involved in its esterase-like activity [21]. Catalytic activity of HSA toward *p*-NPA was investigated to know the involvement of these residues in the binding of HA to HSA. The kinetic constants (*K*_m_ and *V*_max_) were obtained by fitting the initial rates to Michaelis–Menten equation using Graph-Pad Prism, version 5.0, software as shown in Figure S1. Further, the reciprocal of substrate concentration against reciprocal of respective product formation rate are plotted as Lineweaver-Burk plot (Figure 6). The obtained values for all the kinetic parameters are listed in Table 4. The activity of HSA toward *p*-NPA gives *K*_m_ and *V*_max_ equal to 59.36±05.55 µM and 0.16±0.01 µMs^−1^ respectively whereas in presence of HA, *V*_max_ remain same while *K*_m_ increases. This indicates that the HA inhibits the esterase-like activity of HSA competitively with *K*_i_ equals to 21.68 µM and hence Arg410 and Tyr411, of drug binding site II of HSA are involved in binding of HA.

**Table 4:** Kinetic parameters for the hydrolysis of *p*-NPA by HSA.

HSA:HA	K_m_ (µM)	V_max_ (µM s^−1^)	10^5^×*k*_cat_/K_m_ (µM^−1 ^s^−1^)
1∶0	59.36±05.55	0.16±0.01	17.95±1.69
1∶0.5	93.50±04.52	0.16±0.02	11.40±0.55
1∶1	109.5±07.25	0.16±0.01	09.74±0.64
1∶2	126.20±10.16	0.16±0.02	08.45±0.68
1∶3	153.90±09.50	0.16±0.01	06.93±0.42

### Molecular Docking

**Figure 7:**
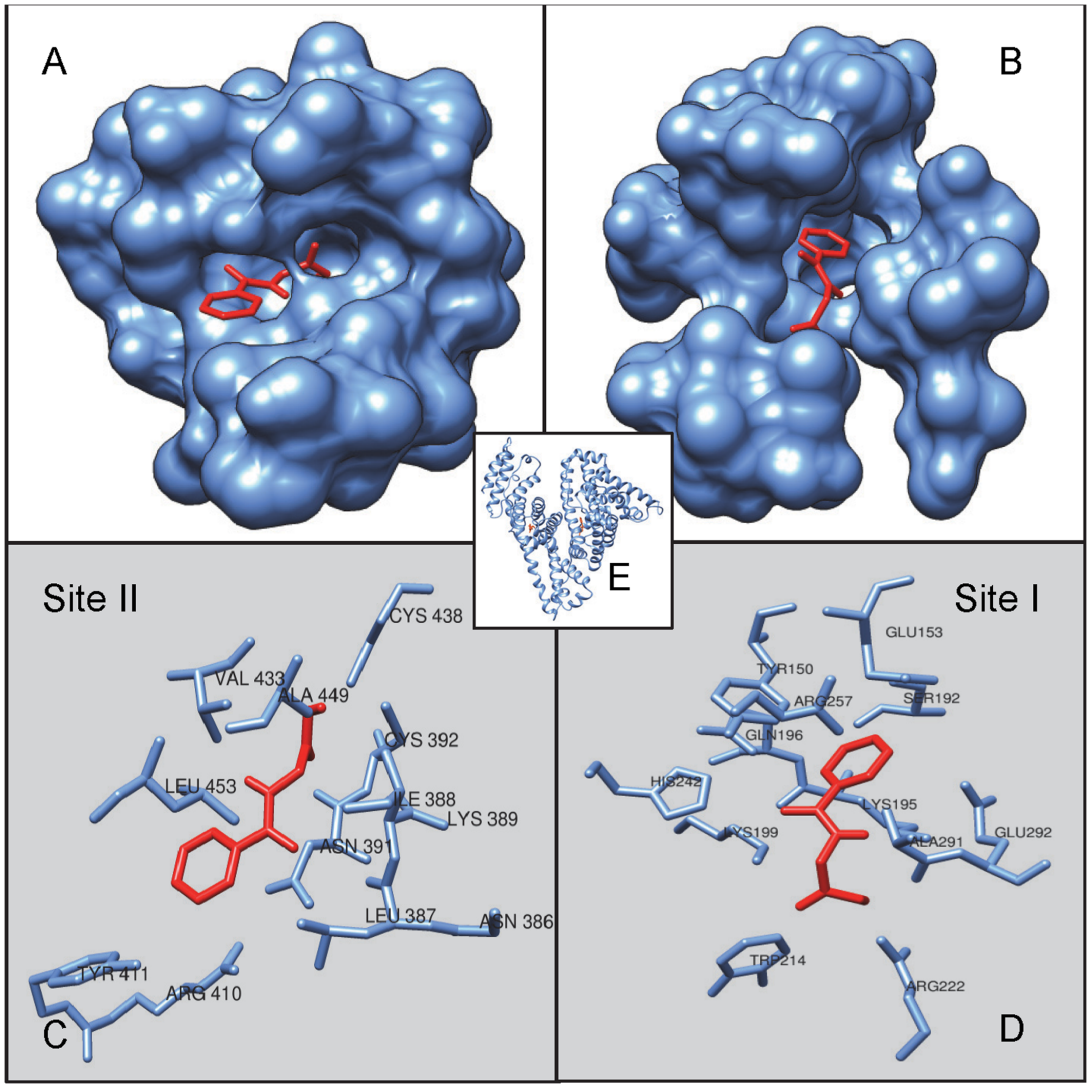
Molecular docking of HA and HSA. **(A)** Molecular surface representation of docked HA in a site II (A) and site I (B) of HSA. Cartoon representation of residue of HSA site II (C) and site I (D) interacting with HA.

The molecular docking has been employed to further understand the interaction of HA and HSA. The HSA comprises of three homologous domains (I–III): I (residues 1–195), II (196–383), III (384–585), each domain comprises subdomains that posses common structural motifs. The principal regions of ligand binding to HSA are located in hydrophobic cavities in subdomains IIA and IIIA, which are consistent with Sudlow sites I and II, respectively [12]. In the present study, Autodock Vina program is applied to calculate the possible conformation of the HA that binds to the protein. The best energy ranked results are summarized in Table 5 and Figure 7, which shows that HA binds to both sites of HSA. Figure 7 B & D show that HA more favourably fit in the hydrophobic cavity in subdomains IIIA, that corresponds to site II, with ΔG and *K*_b_ of −5.9 kcal mol^−1^, 2.12×10^4^ respectively. The Leu387, Ile388, Asn391, Cys392, Leu407, Arg410, Tyr411, Leu430, Val433, Cys438, Ala449 and Leu453 of site II were involved in hydrophobic interaction. However as shown in Figure 7 A & C, HA also fit in cavity of the subdomains IIA, that corresponds to site I, with ΔG and *K*_b_ of −5.6 kcal mol^−1^ and 1.28×10^4^ respectively. The HA interacts hydrophobically with Lys199, Arg222, Tyr150, Glu153, Ser192, Lys195, Gln196, Trp214, His242, Arg257, Ala291 and Glu292 of site I. Moreover, HA forms two hydrogen bonds with Lys199 having bond length of 2.05Å, 2.16Å and one with Arg222, having bond length of 2.36Å. Thus, HA binds to site II with the high affinity binding site whereas with relatively low affinity to site I. For site II, values of Δ*G* obtained by docking are very close to the calorimetrically obtained values whereas, slightly different for site I. The probable explanation for this is that docking was based upon the static and fixed X-ray crystals structure of protein where protein significant structural freedom is not allowed to acquire different conformations upon ligand binding. Unlikely, the calorimetry results are based upon full freedom in the structural flexibility of the protein in aqueous system [25], [43]. Thus, structural rearrangements observed in the HSA that occurred upon HA binding in solution, is a plausible cause of this difference. Further, docking is based on some crucial approximations including the limited number of ligand positions in the trial and omission of protein dynamics. Therefore, molecular docking in this study yields useful information about the specific residues of HSA involved in the interactions with the HA for better understanding of protein-ligand interaction at the molecular level.

**Table 5:** Molecular docking results of HSA-HA interaction.

Binding site	Amino acid	Forces involved	Δ*G* (kcal mol^−1^)	*K*_b_ (M^−1^)
Site I	Lys199	H-bonding and Hydrophobic	−5.6	1.28×10^4^
	Arg222	H-bonding and Hydrophobic		
	Tyr150	Hydrophobic		
	Glu153	Hydrophobic		
	Ser192	Hydrophobic		
	Lys195	Hydrophobic		
	Gln196	Hydrophobic		
	Trp214	Hydrophobic		
	His242	Hydrophobic		
	Arg257	Hydrophobic		
	Ala291	Hydrophobic		
	Glu292	Hydrophobic		
Site II	Leu387	Hydrophobic	−5.9	2.12×10^4^
	Ile388	Hydrophobic		
	Asn391	Hydrophobic		
	Cys392	Hydrophobic		
	Leu407	Hydrophobic		
	Arg410	Hydrophobic		
	Tyr411	Hydrophobic		
	Leu430	Hydrophobic		
	Val433	Hydrophobic		
	Cys438	Hydrophobic		
	Ala449	Hydrophobic		
	Leu453	Hydrophobic		

### Binding of HA to HSA Affect Hemodialysis

HA is formed primarily from aromatic amino acids by gastrointestinal flora or may be directly taken as preservatives from food and beverages [7]. In patients with end-stage renal disease, excretion through kidney is hampered and consequently, the concentration of HA increases to values higher than 247±112 mg/L [8]. So it is believed that it may bind to the HSA and thus its removal through hemodialysis may hamper. Thus to confirm, we examine the binding energetics and amino acid involved in binding of HA to HSA. From above studies, it is found that, HA binds with high affinity to site II while with relatively low affinity to site I of HSA via hydrogen bonding, electrostatic and hydrophobic interaction. Therefore it is confirmed that it bounds to HSA and thus it elimination may be hampered through hemodialysis. Moreover, Arg410 and Tyr411 are involved in binding of HA to site II of HSA, that are also crucial for esterase-like activity of HSA. Thus, HA also impinges esterase-like activity of HSA.

### Conclusions

The present work reports the interaction of HA, a uremic toxin, to HSA. Results indicated that it markedly binds to both drug binding sites of HSA; however binding at site II is relatively more. Further, the quenching mechanism of fluorescence of HSA by HA is a static procedure and their binding is a spontaneous, enthalpically driven, entropically opposed process that involves hydrogen bonding, electrostatic, and hydrophobic interaction. Since it binds to HSA, so its elimination through hemodialysis may hinder. Moreover, it increases the thermostabilty of HSA on binding and inhibits the esterase-like activity of HSA in a competitive manner.

## Supporting Information

Figure S1 **Michalies-Menten plot of HSA for** ***p*** **-NPA at HSA: HA ratio of 1∶0, 1∶0.5 1:1, 1∶1.5, and 1∶2.**
(TIF)
