# Supplementary figures and images for: A Comprehensive Insight into Binding of Hippuric Acid to Human Serum Albumin: A Study to Uncover Its Impaired Elimination through Hemodialysis
Source: PLoS One. 2013 Aug 9;8(8):e71422. doi: 10.1371/journal.pone.0071422 (PMC3739763; doi:10.1371/journal.pone.0071422)

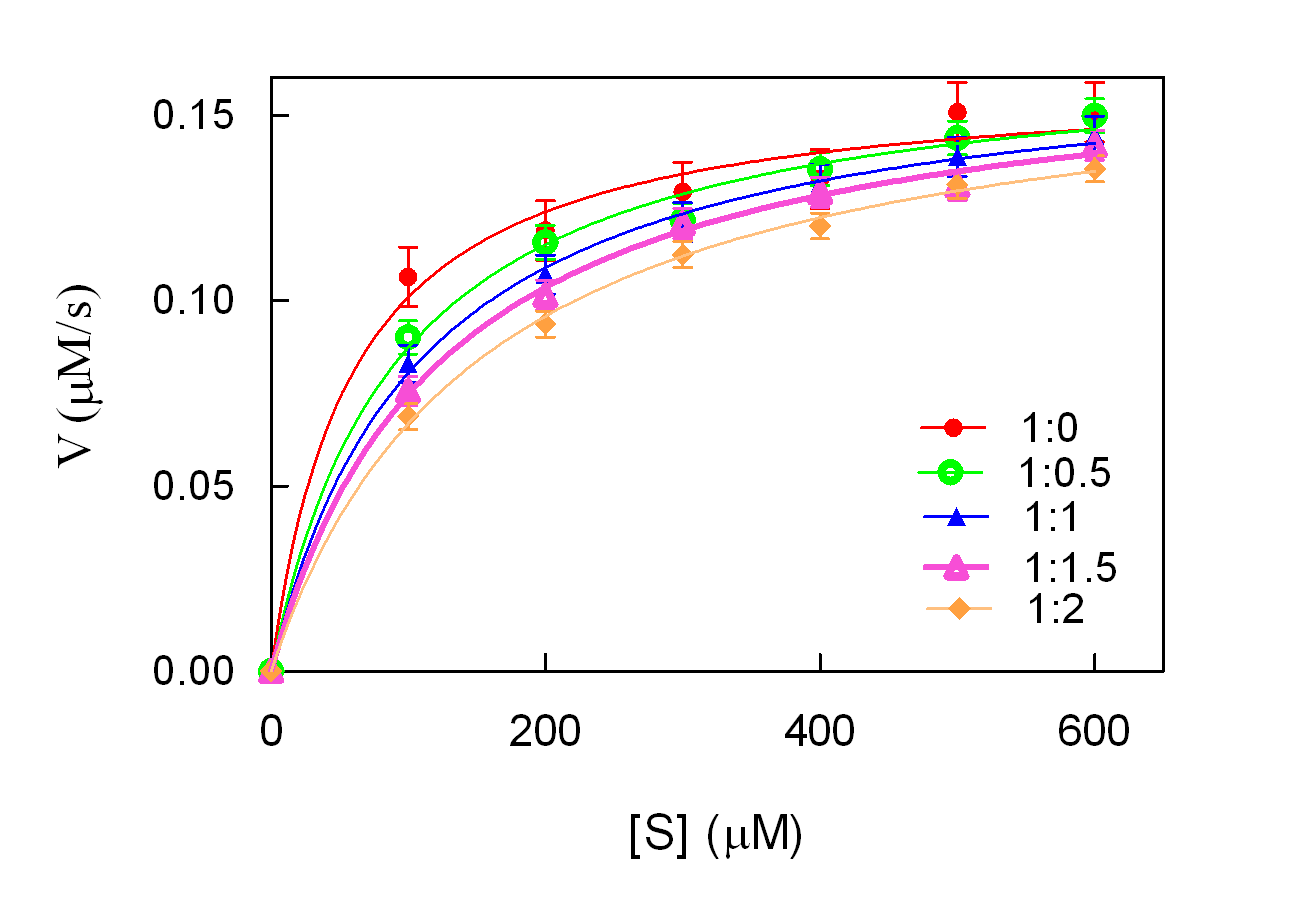

Supplement: Figure S1 — Michalies-Menten plot of HSA for p -NPA at HSA: HA ratio of 1∶0, 1∶0.5 1:1, 1∶1.5, and 1∶2. (TIF) [file pone.0071422.s001.tif]
